# Supplementary material for: Bi-Temporal Anodal Transcranial Direct Current Stimulation during Slow-Wave Sleep Boosts Slow-Wave Density but Not Memory Consolidation
Source: Brain Sci. 2021 Mar 24;11(4):410. doi: 10.3390/brainsci11040410 (PMC8064104; doi:10.3390/brainsci11040410)
Supplement: Supplementary file 1 [file brainsci-11-00410-s001.zip › brainsci-1090048-supplementary.pdf]

Supplementary Tables

Table S1. SPM output list of significant clusters that are functionally connected with the seed mask (i.e. bilateral hippocampus).

cluster-level		peak-level				
$p_{FWE-corr}$	k_E	$p_{FWE-corr}$	T	x	y	z
< 0.001	325290	< 0.001	33.72	-26	-16	-18
< 0.001	1643	< 0.001	15.86	-42	-70	36
< 0.001	1322	< 0.001	13.28	52	-70	34
< 0.001	376	< 0.001	12.10	-14	-88	-40
< 0.001	486	< 0.001	12.01	14	-86	-38
< 0.001	881	< 0.001	11.41	22	32	44
< 0.001	759	< 0.001	10.78	-6	-50	-42
< 0.001	346	< 0.001	10.53	42	-70	-36
< 0.001	5085	< 0.001	9.95	36	-26	56
< 0.001	94	< 0.001	7.93	44	-20	22
< 0.001	99	< 0.001	7.65	-46	-70	-38
< 0.001	38	< 0.001	6.62	0	-22	-20
< 0.001	46	0.001	6.26	0	-34	-24

Table S2. Participants' sleep habits (self-reports, retrospectively assessed using the PSQI) over four weeks of time prior the first recording session (excluding the night prior the first recording that was restricted to 5 hours). Sleep anticipation represents the difference between usual go to bed time and start time of the sleep EEG in minutes.

Parameter	mean	sd	min	max
Go to bed time (hh:mm)	23:34	00:36	22:15	00:30
Sleep-onset time (min)	12.222	7.760	2.5	30
Sleep duration (hh:mm)	07:08	0:44	6:00	8:00

	sham[1]		tDCS		tDCS vs. sham	
	mean	sd	mean	sd	Z	p
Sleep anticipation (min)	160.94	44.99	162.33	40.66	-0.283	.777

[1]Mean and standard deviation averaged across all 18 participants are reported separately for the sham and the tDCS condition, where applicable. Significance of the effect of stimulation (tDCS vs. sham) was tested by using non-parametric Wilcoxon-test.
